# Is cystourethroscopy a crucial preoperative step in severe and complex types of hypospadias?

**DOI:** 10.3389/fsurg.2023.1202539

**Published:** 2023-06-23

**Authors:** Ahmed Oshiba, Mostafa Kotb

**Affiliations:** Department of Pediatric Surgery, Alexandria Faculty of Medicine, Alexandria, Egypt

**Keywords:** children, cystourethroscopy, difficult catheterization, proximal hypospadias, hypertrophied verumontanum

## Abstract

**Background and objectives:**

Proximal hypospadias is considered the most severe subtype of the hypospadias spectrum and represents approximately one-fifth of the total cases. It is well-evidenced by many studies that the rate of postoperative complications following the repair of this complex subtype is significantly higher when compared to the distal variants. Few reports described the proximal hypospadias from the other perspective which is the preoperative one. Most pediatric surgeons notice an unexplained incidence of lower urinary tract infection and occasional difficulty of urinary catheterization in those children. This sometimes requires the use of additional measures such as the use of urethral sounds, filiforms and followers, and even catheterization under anesthesia. The aim of the work is to evaluate the role of preoperative cystourethroscopy in detecting associated anomalies in cases of proximal and severe hypospadias.

**Materials and methods:**

This prospective study was conducted in the pediatric surgery unit at Alexandria Faculty of Medicine between July 2020 and December 2021 and included all children with severe grades of hypospadias. After thorough evaluation, all children underwent cystourethroscopy just before the procedure. Any abnormalities in the urethra, urinary bladder, or ureteric openings were recorded if present. Finally, the definitive operation was performed as per schedule.

**Results:**

Fifty-two patients (41 fresh and 11 redo patients) with a median (range) age at presentation of 5 (1–16) years were enrolled in this study. The intraoperative cystourethroscopy was done in all of the patients. Significant abnormal findings were recorded in 32 patients (61.5%), while the other 20 patients (38.5%) were revealed to be normal. The most common abnormal findings were dilated prostatic utricle opening and hypertrophied verumontanum (in 23 and 16 cases, respectively).

**Conclusion:**

Although most of the associated anomalies with proximal hypospadias are asymptomatic, cystourethroscopy is better used owing to a high incidence of these anomalies. This can facilitate an early diagnosis as well as early detection and intervention at the time of repair.

## Introduction

Proximal hypospadias is defined by the presence of urethral meatus at the penoscrotal junction after the procedure of penile degloving in the operating room. It is considered the most severe subtype of the hypospadias spectrum and represents approximately one-fifth of the total cases (1). It is well-evidenced by many studies that the rate of postoperative complications following the repair of this complex subtype is significantly higher when compared to the distal variants. The most common of which are urethrocutaneous fistula, urethral stenosis, and glans dehiscence. Others include stricture, urethral diverticulum, persistent chordee, and unsatisfactory cosmetic appearance (2).

Few reports described the proximal hypospadias from the other perspective which is the preoperative one. Most pediatric surgeons notice an unexplained incidence of lower urinary tract infection and occasional difficulty of urinary catheterization in those children. This sometimes requires the use of additional measures such as the use of urethral sounds, filiforms and followers, and even catheterization under anesthesia (3). This could be possibly attributed by the results of Devine et al. who noted a 14% incidence of enlarged prostatic utricle in patients with proximal hypospadias examined by a cystourethroscopy just before the procedure of hypospadias repair (4). The aim of the work is to evaluate the role of preoperative cystourethroscopy in detecting associated anomalies in cases of proximal and severe hypospadias.

## Materials and methods

This prospective study was conducted in the pediatric surgery unit at Alexandria Faculty of Medicine between July 2020 and December 2021. We included all children with severe grades of hypospadias, i.e., proximal penile, penoscrotal types, as well as patients with scrotal hypospadias and bifid scrotum. Anterior types of hypospadias (mid-penile, subcoronal, coronal, and glanular types) were excluded from our study. After the approval of the ethical committee of our institute, the enrolled patients were thoroughly examined clinically and underwent a radiological investigation using ultrasonography to detect any upper or lower urinary tract anomalies. On the day of the surgery, the patients underwent cystourethroscopy just before the procedure. Any abnormalities in the urethra, urinary bladder, or ureteric openings were recorded if present. Finally, the definitive operation was performed as per scheduled.

## Results

Fifty-two patients (41 fresh and 11 redo patients) with a median (range) age at presentation of 5 (1–16) years were enrolled in this study. Abnormal location of the urethral meatus was the urinary complaint in all the patients. Thirty-two patients had associated cryptorchidism, two patients presented with day and night urinary incontinence, and one patient with recurrent orchitis, while a previous history of low anorectal malformation was seen in one patient. Chordee was present in all patients with variable degrees of severity. The types of hypospadias encountered were as follows: 25 patients had proximal penile, 15 had penoscrotal, and 12 had scrotal hypospadias with bifid scrotum subtype (Table 1).

**Table 1:** Types of hypospadias.

Type of hypospadias	Number (*n*)
Proximal penile	25
Penoscrotal hypospadias	15
Scrotal hypospadias with bifid scrotum	12

**Figure 1:**
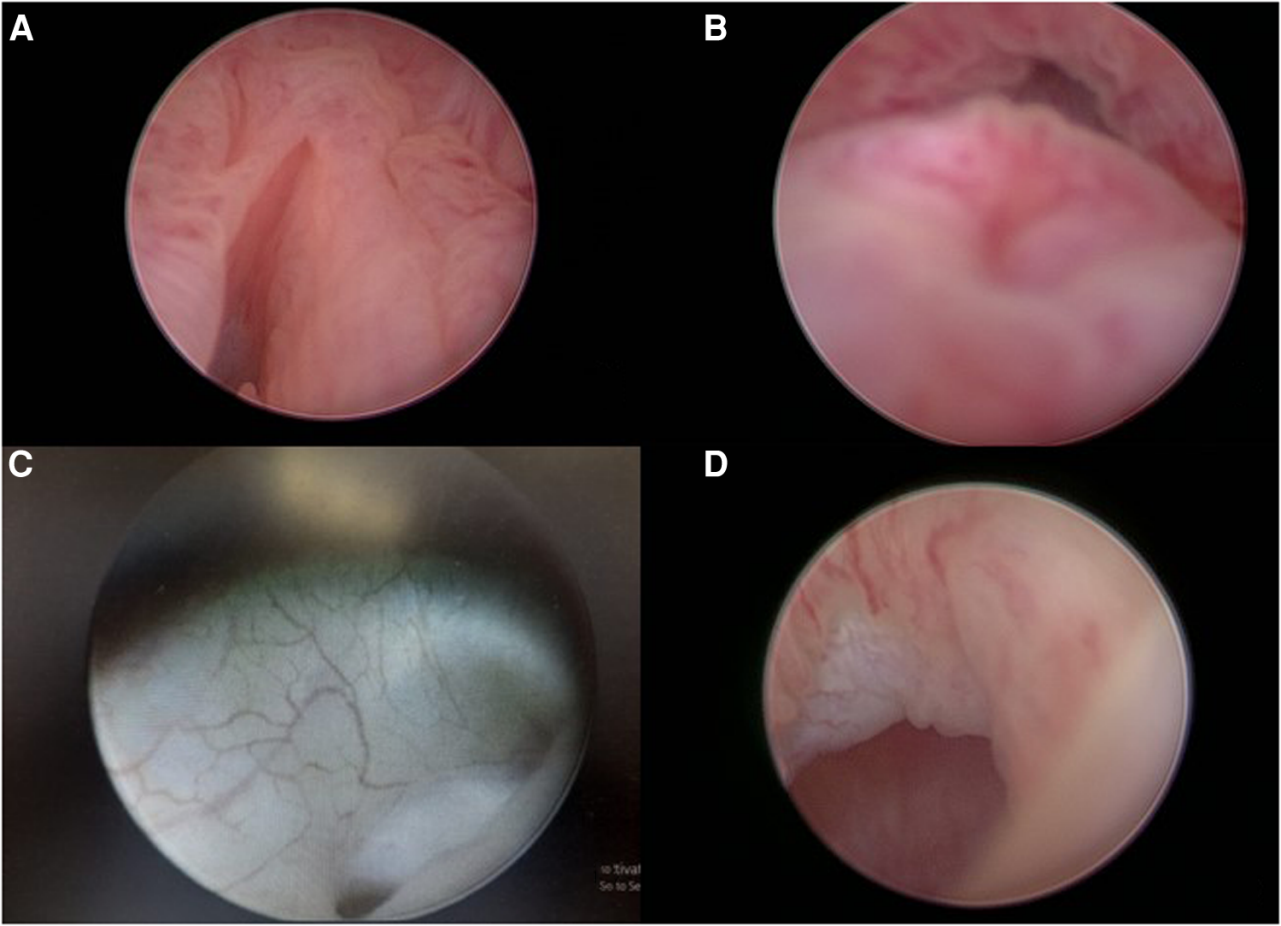
Cystourethroscopic findings. (**A**) Dilated utricle opening. (**B**) Hypertrophied verumontanum. (**C**) Refluxing left ureter. (**D**) Opening of the utricle cyst.

The intraoperative cystourethroscopy was done in all of the patients. Significant abnormal findings were recorded in 32 patients (61.5%) (Figure 1), while the other 20 patients (38.5%) were revealed to be normal. Abnormal findings were in the form of dilated prostatic utricle opening in 23 patients, hypertrophied verumontanum in 16 patients, absent verumontanum and incompetent bladder neck in 2 patients, Müllerian remnants (vaginal pouch) in 2 patients, dilated unilateral refluxing ureter in 2 patients, dilated bilateral refluxing ureters in 1 patient, huge utricle cyst with ectopic inserted vas deferens in 1 patient, and posterior urethral diverticulum post posterior sagittal anorectoplasty (PSARP) in 1 patient (Table 2).

**Table 2:** Summary of the preoperative cystourethroscopic findings.

Cystourethroscopy findings	Number (*n*)
Dilated prostatic utricle opening	23
Hypertrophied verumontanum	16
Absent verumontanum and incompetent bladder neck	2
Müllerian remnants (vaginal pouch)	2
Dilated unilateral refluxing ureter	2
Dilated bilateral refluxing ureters	1
Huge utricle cyst with ectopic inserted vas deferens	1
Posterior urethral diverticulum post PSARP	1

Three cases had associated vesicoureteric reflux and started chemoprophylaxis, two of which were injected with Deflux after a while due to recurrent breakthrough urinary tract infections. In another patient, a huge utricle cyst with ectopic inserted vas deferens was found and attempted by using the Rendez-vous technique (a combination of laparoscopy and cystoscopy); however, the procedure was terminated after finding both vasa differentia ectopically inserted in the cyst. Patients with huge utricle cyst and Müllerian remnants underwent genetic karyotyping and were found to be XY disorders of sex differentiation (XY DSD). The posterior diverticulum post PSARP was excised through a perineal approach in a separate session. Urethroplasty was done subsequently, and 12 patients were resistant to urinary catheterization and were managed by sounds in 6 patients and by cystoscopy guidance in another 6.

## Discussion

Inguinal hernia and cryptorchidism are the most common anomalies associated with hypospadias (7%–13% of patients), being more frequent in the more proximal subtypes (5). Several reports found a high incidence of upper urinary tract anomalies in association with hypospadias, and they believe that routine upper tract screening is necessary. Nevertheless, these anomalies were found to be more evident in penoscrotal and perineal types as well as those associated with other extra-urinary abnormalities (5). In a study conducted by Khuri et al., it was found that an incidence of significant upper tract anomalies was 7%, 13%, and 37% when one, two, or three other organ system abnormalities are associated with hypospadias, respectively. On the other hand, should the proximal hypospadias present in an isolated form, the incidence of associated upper tract anomalies is as low as below 5% (6). In the more distal types, unless other organ anomalies are present, the incidence is similar to that in the general population (6–8). Thus, amid assessing a case of hypospadias, upper urinary tract screening with renal ultrasonography and/or voiding cystourethrogram (VCUG) is better performed in patients with proximal subtypes (i.e., penoscrotal and perineal), syndromic patients along with some patients such as symptomatic children, and those with a strong family history of urinary tract abnormalities (9).

Few studies described the incidence of lower urinary tract anomalies in such complex forms of hypospadias. The earliest study was conducted by Devine et al. on 44 children presented with proximal hypospadias to study the utricular configuration using a cystourethroscopy that was immediately done before the operation of repair (4). Patients who were included in this study were proximal penile, penoscrotal, and perineal subtypes and were found to have an abnormally enlarged utricle in 0%, 10%, and 57%, respectively. They concluded that this anomaly can be a manifestation of delayed Müllerian duct regression or decreased androgenic stimulation of the urogenital sinus (4).

In a more recent and broad study by Gupta et al., a total of 60 patients from all types of hypospadias underwent a comprehensive urinary system assessment using an ultrasound, voiding study, and preoperative cystourethroscopy (10). There was an overall occurrence of positive findings on cystourethroscopy in around one-third of the cases (35%). Seven percent of these anomalies were among the anterior group (glanular, coronal, and subcoronal), 20% in the middle group (distal, middle, and proximal), and 8% in the penoscrotal group. The most frequently encountered anomalies included trabeculated bladder, ectopic ureteric orifice, hypertrophied verumontanum, vertical ridges in posterior urethra, and prominent cristae colliculi along with other anomalies (10).

In this study, we attempted to be more specific by highlighting the cystourethroscopic findings in the more complex hypospadias cases only (i.e., proximal hypospadias, penoscrotal hypospadias, and scrotal hypospadias with bifid scrotum). The rationale was to understand the cause of repeated urinary tract infection as well as difficulty in catheterization in those children which could possibly be due to an anatomical aberration. Approximately 60% of our patients were found to have significant cystourethroscopic findings. The most common findings were dilated prostatic utricle opening in 23 cases (44%) and hypertrophied verumontanum in 16 cases (25%). The former was in agreement with Devine et al. who found a high incidence of such anomaly with the more proximal and complex forms of hypospadias (4). Other anomalies encountered with lower frequencies were refluxing ureters, huge utricle cyst with ectopic inserted vas deferens, and posterior urethral diverticulum post PSARP. Again, most of these findings were found in the study performed by Gupta et al. with different frequencies (10).

The use of preoperative cystoscopy enabled early detection and intervention, i.e., urethroplasty and the associated anomaly. In our study, this happened in three different occasions. Three patients had associated vesicoureteric reflux and started chemoprophylaxis, two of whom were injected with Deflux after a while due to recurrent breakthrough urinary tract infections. The third patient presented with repeated urinary tract infections and recurrent orchitis and was found to have a huge utricle cyst. This was determined by using the Rendez-vous technique (a combination of laparoscopy and cystoscopy) and was described in a separate report (11).

To sum up, although most of the associated anomalies with proximal hypospadias are asymptomatic, cystourethroscopy is better used owing to a high incidence of these anomalies. This can facilitate an early diagnosis as well as early detection and intervention at the time of repair and could be helpful in the trial of catheterizing the difficult cases in order to avoid the creation of a false passage during catheter insertion.

## Data Availability

The original contributions presented in the study are included in the article, further inquiries can be directed to the corresponding author.
